# A “Catastrophic” Heparin-Induced Thrombocytopenia

**DOI:** 10.1155/2020/6985020

**Published:** 2020-04-13

**Authors:** D. Barcellona, M. Melis, G. Floris, A. Mameli, A. Muroni, G. Defazio, F. Marongiu

**Affiliations:** ^**1**^ Haemostasis and Thrombosis Departmental Unit, AOU Cagliari, Department of Medical Science and Public Health, University of Cagliari, Cagliari, Italy; ^**2**^ Neurology Complex Unit, AOU Cagliari, Department of Medical Science and Public Health, University of Cagliari, Cagliari, Italy; ^**3**^ Internal Medicine Complex Unit, AOU Cagliari, Department of Medical Science and Public Health, University of Cagliari, Cagliari, Italy

## Abstract

**Background:**

Heparin-induced thrombocytopenia (HIT) is a transient, antibody-mediated thrombocytopenia syndrome that usually follows exposure to unfractioned heparin (UFH) or low-molecular-weight heparin (LMWH). In contrast to other pathological conditions which lead to thrombocytopenia and bleeding complications, HIT results in a paradoxical prothrombotic state. It is caused by antibodies directed to complexes containing UFH or LMWH and a self-platelet protein: the platelet factor 4 (PF4). The heparin-PF4 immune complex leads to activation of platelets, monocytes, and endothelial cells which release procoagulant proteins and tissue factor with subsequent blood coagulation activation. *Case Report*. We describe the case of a woman undergone to knee replacement and affected by urosepsis who developed a HIT after exposure to enoxaparin. The thrombotic burden was very impressive involving the arterial and venous cerebral vessel and the venous pulmonary, hepatic, and inferior legs vascular beds. The patient was successfully treated with fondaparinux without recurrent thrombosis or bleeding. The clinical scenario could be named “catastrophic HIT” like the catastrophic antiphospholipid syndrome since they have a similar pathogenetic mechanism involving both platelets and monocytes procoagulant activities and a similar clinical manifestation with a life-threatening multiple arterial and/or venous thromboses.

**Conclusion:**

Patients presenting with HIT could show a very impressive thrombotic burden resembling to that of the catastrophic antiphospholipid syndrome. A careful differential diagnosis should be made towards other pathological conditions which lead to thrombocytopenia to avoid an unnecessary and potentially harmful platelet transfusion. Although fondaparinux is off-label, its use in patients with HIT is simple and seems to be effective.

## 1. Case Presentation

We describe the case of a woman, 63-years-old, who developed a severe heparin-induced thrombocytopenia with arterial and venous thrombosis.

On April 2019, the patient had undergone a knee replacement surgery for her left knee osteoarthritis and was discharged from the hospital with enoxaparin 40 mg, subcutaneously, once a day, as a thromboembolic prophylaxis. No laboratory checks had been recommended for platelets count monitoring.

Fifteen days later, the patient was admitted to our Emergency Department for an episode of hyperpyrexia (39.5°C), headache, right hemiplegia, and an episode of consciousness alteration with cloned tremors in the lower right limb and abdomen, lasting about two minutes. The brain computerized tomography (CT) scan was negative, but the magnetic resonance imaging (MRI) showed an acute ischemia of the postcentral and precentral rounds of the left brain hemisphere and another subacute ischemic lesion of the right parietal region.

The laboratory findings were all in the normal range except for modest anemia (Hb = 10.6 gr/dl) and thrombocytopenia (43000 × 10^9^/L) and high D-Dimer (D-D) level (34.800 *η*g/L).

The patient was transferred to the Neurology Department.

The clinical history was negative for arterial thrombosis risk factors. In particular, she had no hypertension, diabetes, or hypercholesterolemia, she was not overweight, and she had never smoked or taken estroprogestins. The clinical examination highlighted a severe aching in the right hypochondrium without increase in liver and spleen size. No signs of mucous-cutaneous bleeding were present.

The electrocardiogram and the echocardiography showed a sinus rhythm with a nondilated left atrium and a normal ejection fraction (65%) of the left ventriculum thus excluding an atrial fibrillation or other causes of cardioembolism. The ultrasound of the supra-aortic trunks was also normal. The CT scan of the thorax surprisingly showed a pulmonary embolism with a minus imaging on the main branch of the left pulmonary artery and its segmentary and subsegmentary branches of the dorsal pulmonary region. The patient was completely asymptomatic. The CT scan of the abdomen highlighted a minus imaging on some segmentary brunches of the right suprahepatic vein that may justify the pain in the right hypochondrium to the liver palpation. The portal vein was normal and the aorta was free from atherosclerotic lesions. Because of the pulmonary embolism, a venous ultrasound of the inferior limbs was performed showing a nonrecent left superficial femoral and popliteal veins thrombosis.

The platelet count was confirmed low when the laboratory test was repeated using a test tube containing citrate instead of ethylenediaminetetraacetic acid.

The clinical suspicion of a HIT diagnosis was supported by the use of low-molecular-weight heparin, a normal platelet count (226000 × 10^9^/L) before the orthopedic surgery, no bleeding and the exclusion of a thrombocytopenia due to splenomegaly, pseudothrombocytopenia, and autoimmune thrombocytopenia. The pretest scoring system for HIT diagnosis, the 4 T's score [1], was of 8 points since the platelet count falls more than 50%, the onset of the platelet fall was within 15 days, several new arterial and venous thrombosis were objectively diagnosed, and no other causes for thrombocytopenia were found. The anti-platelet factor 4 (anti-PF4) antibodies determination (HIT-Ab (PF4–H), Werfen, Barcelona, Spain) showed a title of 1.5 U/ml (normal value 0.0–1.0 U/ml). The enoxaparin was immediately stopped, and a treatment with fondaparinux 5 mg, subcutaneously, once a day was given. Fondaparinux was used at subtherapeutic dosage, and the patient weight was 70 kilograms, since a new brain MRI showed a haemorragic evolution of the acute ischemic cerebral lesion.

Three days after the admission, the patient had a new episode of hyperpyrexia and the chemical-physical urine test showed the presence in the sediment of erythrocytes, leukocytes, bacteria, and albuminuria, so ciprofloxacin 400 mg bid IV was started. The urinoculture highlighted a multiresistant *Escherichia coli* infection. The erythrocytes sedimentation velocity (30 mm, normal value 0–12 mm), the C-reactive protein (39.6 mg/dl, normal value 0–5 mg/dl), and the procalcitonin (0.62, normal value <0.5) were all above the normal range. The fibrinogen level was quite low, 93 mg/dl, and the prothrombin time (PT) was modestly prolonged, 1.43 INR. The laboratory and clinical findings suggested an urosepsis. The ciprofloxacin was stopped, and a meropenem therapy was started on the basis of the antibiogram. Fresh frozen plasma at the dosage of 20 ml/kg was administered.

Because of the persistence of the headache despite the pain-relieving therapy, a brain MRI with contrast medium was performed. The exam showed a superior sagittal, rectum, and bilateral transverse cerebral sinus thrombosis.

After replacing enoxaparin with fondaparinux, the platelet count was above 50000 × 10^9^/L in 2 days and completely recovered in 5 days (161000 × 10^9^/L). After 25 days, the patient was transferred to the rehabilitation ward for her right hemiplegia. At 6 months of follow-up, a new CT total body scan with contrast medium showed parieto-occipital cerebral bilateral ischemic outcomes, a small imagine of minus in the left transverse cerebral sinus, and a complete recanalization of the pulmonary vascular bed and the right suprahepatic vein. The venous ultrasound of the inferior limbs showed a complete recanalization of the left superficial femoral and popliteal veins. The anti-platelet factor 4 (anti-PF4) antibody determination was 0 U/ml.

## 2. Discussion

HIT is a potential life-threatening disease characterized by a decrease in platelet count and thrombosis after heparin exposure. Two different types of HIT are recognized: (1) HIT type I, first described in 1989 [2], that is a mild thrombocytopenia of early onset not associated with an increased risk of thrombosis; (2) HIT type II, first reported in 1958 [3], that occurs after 7–14 days, characterized by more severe clinical manifestations such as venous or arterial thrombosis. A third type of HIT was described by Warkentin et al. [4], the so called spontaneous HIT, in which the formation of platelet-activating anti-PF4/heparin antibodies can occur without previous heparin exposure. We describe the case of a woman who developed a severe type II HIT characterized by a very important thrombotic burden.

The patient had at least two associated risk factors: the recent orthopedic surgery and urosepsis. Orthopedic surgery was the most important risk factor for HIT-associated thrombosis in 408 patients with clinically suspected HIT in the paper by Greinacher et al. [5] who demonstrated that the risk for HIT-associated thrombosis increases of about 5 folds.

During bacterial infections, platelets are activated and release positively charged PF4 which can bind to the negatively charged lipopolysaccharide (LPS), a component of Gram-negative bacterial cell surface. It has been demonstrated [6] that the interaction of LPS with platelet-PF4 can induce epitopes, resembling those on PF4-heparin complexes that can bind human anti-PF4/heparin antibodies. Krauel et al. also showed that a mutant bacterium shows increasingly enhanced PF4 binding activity. Moreover, Maharaj and Chang [7] have recently shown that the rate of anti-PF4/heparin antibodies is increased in patients hospitalized with bacterial sepsis.

Our patient had undergone a left knee replacement surgery and showed an urosepsis provoked by a multiresistant *Escherichia coli*. The patient presented with a low platelet count, a high D-D level, and a prolonged PT. At first, fibrinogen level was falsely normal, since higher values were expected due to sepsis, and in day 2, fresh frozen plasma at a dosage of 20 ml/kg was administered to the patient since fibrinogen dropped to 93 mg/dl. This clinical picture, to be attributed to urosepsis, could resemble a disseminated intravascular coagulation but no mucous-cutaneous bleeding was evident. It is also important to note that the patient was not treated with platelet transfusion despite platelet count falls more than 50%. It has been demonstrated [8] that platelet transfusions in patients admitted with HIT were associated with a 3- and 5-fold higher risk of arterial thrombosis and all-cause mortality, respectively. Therefore, in the presence of a thrombocytopenia, a careful differential diagnosis should be made to avoid platelet transfusion that in HIT should deserve only to invasive procedures, surgeries, or life-threatening bleeding.

We hypothesize that the severe thrombotic clinical picture of our patient could be the result of the association of these two risk factors, orthopedic surgery and urosepsis, that extremely enhanced the blood coagulation activity. To our knowledge, this is the first case report that describes a patient with arterial and venous thrombosis involving cerebral, pulmonary, hepatic, and lower limbs vascular beds. This clinical scenario could be named “catastrophic HIT,” like the catastrophic antiphospholipid syndrome, since they have a similar pathogenetic mechanism involving both platelets and monocytes procoagulant activities [9] and a similar clinical manifestation with a life-threatening multiple arterial and/or venous thromboses.

Several drugs can be administered to patients affected by HIT-associated thrombosis. Argatroban and bivalirudin are synthetic thrombin inhibitors approved in the USA and Europe for this indication. Both drugs can be administered intravenously and require adjusted doses to maintain partial thromboplastin time at 1.5–3.0 times baseline values [10]. Danaparoid has an indication for HIT-associated thrombosis in Europe and Canada and is a mixture of sulfated glycosaminoglycans that causes a long-acting antithrombin-dependent inhibition of activated factor X (Xa). It can be administered intravenously, and a monitoring of anti-Xa activity should be undertaken only in patients with extreme body weight (<55 kg, >90 kg) [10]. Direct oral anticoagulants (DOACs) and fondaparinux are emerging as HIT treatment options even if there is no regulatory approval for this indication. DOACs were used in 81 patients of whom 2.5% have been reported to develop a recurrent thrombosis. [11, 12].

Fondaparinux is a long-acting inhibitor of factor Xa that, although its off-label status, seems an appropriate anticoagulant to use in HIT patients, as recently shown by Warkentin [13].

We chose to use fondaparinux, since the glomerular filtration rate of our patient was good and the administration of this drug is simple for a neurological ward given that it does not require monitoring and can be administered subcutaneously.

## 3. Conclusion

Patients presenting with HIT could show a very impressive thrombotic burden resembling to that of the catastrophic antiphospholipid syndrome. A careful differential diagnosis should be made towards other pathological conditions which lead to thrombocytopenia to avoid an unnecessary and potentially harmful platelet transfusion. Although fondaparinux is off-label, its use in patients with HIT is simple and seems to be effective.
